# Clinical Trial Schedule of Activities Specification Using Fast Healthcare Interoperability Resources Definitional Resources: Mixed Methods Study

**DOI:** 10.2196/71430

**Published:** 2025-10-20

**Authors:** Andrew Richardson, Patrick Genyn

**Affiliations:** 1 fhir4pharma Hungerford, Berkshire United Kingdom; 2 fhir4pharma Doylestown, PA United States

**Keywords:** Fast Healthcare Interoperability Resources, FHIR, Health Level 7, HL7, graph methods, clinical trials, schedule of activities, interoperability, electronic health records

## Abstract

**Background:**

Clinical research studies rely on schedules of activities (SoAs) to define what data must be collected and when. Traditionally presented in tabular form within study protocols, SoAs are critical for ensuring data quality, regulatory compliance, and correct study execution. Recent efforts, such as the Health Level 7 Vulcan SoA Implementation Guide, have introduced Fast Healthcare Interoperability Resources (FHIR) as a standard for representing SoAs digitally. However, current approaches primarily handle simple schedules and do not adequately capture complex requirements such as conditional branching, repeat cycles, or unscheduled events—features essential for many study designs, particularly in oncology.

**Objective:**

This study aimed to extend SoA representation methods to address these limitations. Specific objectives were to (1) develop methods for defining multiple SoA paths within a single model, (2) specify conditional scheduling requirements, (3) design a human-readable syntax for study specifications, (4) reflect these requirements as FHIR definitional resources, and (5) test bidirectional conversion between graph-based SoA models and FHIR representations.

**Methods:**

Building on previous work, SoAs were modeled using directed graphs in which nodes represented interactions (eg, visits) or activities, and edges defined transitions. Attributes were added to capture timing, conditional rules, and repeatability. Graph-based models were translated into FHIR PlanDefinitions and related resources (ActivityDefinition, ResearchStudy, and ResearchSubject). Extensions to PlanDefinition were developed (soaTimePoint and soaTransition) to store graph-specific attributes. Proof-of-concept models were implemented and tested using Python, NetworkX, pandas, and FHIR Shorthand, with validation conducted through FHIR servers to ensure structural equivalence and information retention.

**Results:**

The graph-based approach successfully modeled multiple paths, unscheduled events, and conditional rules within a single SoA. Edge attributes such as transitionDelay and transitionRule enabled accurate timing calculations and runtime evaluation of permitted paths. Conditional scheduling was expressed using a parameterized syntax interpretable by logic engines. More than 25 study protocols of varying complexity were tested; all could be represented without information loss. The proposed FHIR extensions allowed PlanDefinition resources to fully capture SoA graphs rather than limited tabular forms. Round-trip testing confirmed that the graph models and FHIR resources could be converted without loss of fidelity. The approach also highlighted inconsistencies in some protocol specifications, suggesting its utility for protocol quality assurance.

**Conclusions:**

This study demonstrates that graph-based modeling, combined with targeted FHIR PlanDefinition extensions, enables an accurate and comprehensive representation of clinical study SoAs, including complex scheduling features that are not supported by current standards. These methods improve interoperability, reduce reliance on manual interpretation, and provide a basis for the automated integration of study protocols with electronic health records. While further tooling (eg, FHIRPath and clinical quality language) is needed for operational deployment, this approach offers a more precise and extensible solution for digital protocol implementation.

## Introduction

### Background

Healthcare interoperability standards such as the Fast Healthcare Interoperability Resources (FHIR) [1] are providing new methodological approaches for the collection, collation, and confirmation of clinical research data for both observational studies and trials supporting product regulatory submissions [2-4]. To be successful, clinical research studies require that (1) the correct data are available to answer the research question and (2) these data are collected at the correct times. These are detailed in the study protocol, where the schedule of activities (SoAs), usually in the form of a square table, provides the key data and scheduling requirements. Various recent projects are underway to explore methods for digitizing all or parts of study protocols with FHIR as a key interoperability component (International Council for Harmonization M11, CDISC Unified Study Definitions Model, and Vulcan Utilizing the Digital Protocol) [5,6].

In a previous paper [7], a graph-based minimum viable set of characteristic attributes needed to define a study’s SoA was developed, along with commentary on the range of “variations on the theme” encountered in different clinical study types and therapeutic areas. Operational use cases that depend on the SoA were also considered. The resulting graph representations of an SoA were converted to FHIR PlanDefinitions compliant with the Health Level 7 (HL7) Vulcan clinical study SoA Implementation Guide (IG) [8].

While the current Vulcan SoA IG can model a wide variety of clinical study SoAs, it is recognized that it is principally limited to defining relatively simple SoAs. It works well to convert SoA tables to FHIR resources; however, it does not have methods to define schedules that repeat (cycle), an essential part of most oncology studies. Similarly, it does not offer methods for conditional switching or to select different permitted (multiple) study paths. It may be coerced in some cases to manage specific situations, but this is not ideal when these “fixes” are key scheduling requirements. This can mean that a Vulcan SoA IG–compliant SoA will specify only part of the total scheduling options that a specific study requires. Subsequent use by consuming applications, such as electronic health record (EHR) systems, may or will require additional tooling to implement all study scheduling variations and control.

Considering previous work, this study investigated (1) what attributes or modifications to a graph model are needed to cover the extended use cases outlined earlier and (2) develop a FHIR PlanDefinition representation that can communicate these requirements. Additional tooling—such as FHIRPath expressions, clinical quality language, or system-specific methods (eg, EHR)—will be required to fully implement the FHIR PlanDefinition into the operational workflow of a clinical system.

### This Study

The primary objectives of this study were to (1) develop methods for defining multiple SoA paths within a single graph, (2) develop methods for defining SoA conditional scheduling requirements, (3) develop a human-understandable syntax to support specific study specification, (4) reflect these requirements as FHIR definitional resources, and (5) test and confirm converting the graph representations to FHIR and vice versa

## Methods

### Graphical SoA Definition

#### Overview

The adopted methodological approach was to (1) build on previous work to investigate and develop the necessary attributes required to meet the objectives described earlier and (2) develop and test FHIR resource options to accurately describe and exchange these requirements.

**Figure 1:**
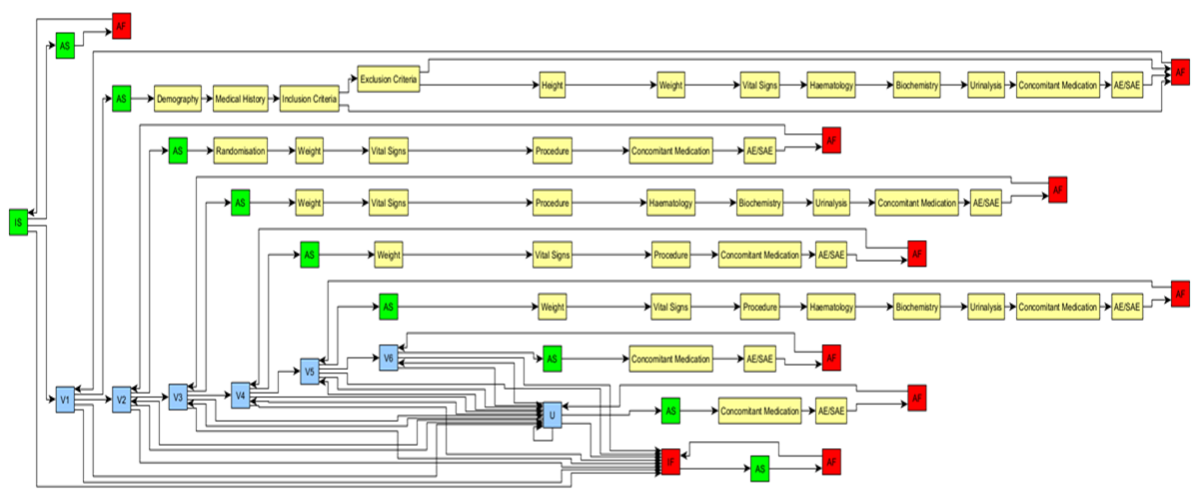
Example study of a schedule of activities directed graph. The schedule of activities has 6 planned visits and 1 unscheduled visit (U; blue). The activities at each visit are shown in yellow. The green and red nodes delineate the start and finish of graph instantiation and the activities to be undertaken contiguously. AS: activity start; AF: activity finish; IS: instantiation start; IF: instantiation finish.

Table 1 shows part of an SoA as might be presented in a protocol, and Figure 1 [7] shows a graphical representation of the primary schedule elements. The minimum set of characteristic attributes required to reflect it in this form is shown in Table 2 (refer to the study by Richardson [7] for more details). Two SoA graph node types are used in this model: “interactions,” modeling study events, visits, or other direct or indirect contacts with research participants and “activities,” defining the tasks required to be undertaken to meet the study objectives. These have a simple and easily understood correspondence with the tabular forms found in protocols. These were used to convert the SoAs into Vulcan SoA IG–compliant FHIR resources [7].

**Table 1:** Example (partial) of a schedule of activities as presented in protocols.

Phase		Screening	Study period					Unscheduled
Visit ID		1	2	3	...^a^	6	U	
Visit timing		–Days 28 to 1	Day 1	Day 7	...	Day 28	As required	
**Activity**								
	Demographics	X						
	Medical history	X						
	Inclusion and exclusion criteria	X		X				
	Vital signs	X	X	X	X	X	X	
	Procedure	X	X					
	...^b^							
	Concomitant medication	X	X	X	X	X	X	
	AE^c^ and SAE^d^	X	X	X	X	X	X	

^a^Additional visit IDs (columns) exist in complete schedules of activities.

^b^Additional activities (rows) exist in complete schedules of activities.

^c^AE: adverse event.

^d^SAE: serious adverse event.

**Table 2:** Schedule of activities graph characteristic attributes^a^.

Attribute		Minimal required attribute		Notes or example			
**Nodes**							
	nodeID		Yes		Universally unique identifier		
	Type		Yes		“Interaction” or “activity”		
	Subtype		No		For example, clinic visit and telephone call		
	Name		Yes		Protocol name of interaction or activity		
	Description		No		Description of interaction or activity		
	plannedTiming		Yes		Schedule timing (D1 etc)		
	referenceTimepoint		Yes		Schedule t(zero)		
	plannedWindow		No		Schedule timing permitted variance		
	plannedDuration		No		Duration of interaction or activity (eg, 24 h)		
**Edges**							
	edgeID		Yes		Universally unique identifier		
	transitionType		No		Timing relationship between nodes		

^a^Adapted from the study by Richardson [7].

#### Multiple SoA Paths

Standard graph methods were used to develop and test the attributes necessary to model and define the routing objectives (use cases) established earlier. The primary considerations were to (1) accurately reflect different study scheduling options using property graph methods and (2) ensure that the resulting models had a user-friendly correspondence to standard clinical trial scheduling concepts. Only methods using directed graphs were considered.

#### Conditional Scheduling

Methods to accurately model the conditional scheduling requirements within a single SoA graph initially used path analysis to identify the set of adjacency matrices required for each scenario. These were then used to develop a specification syntax that could define any specific conditional requirement, which, with appropriate tooling, can be implemented such that any subgraph can be extracted from the primary specification. Consideration was given to ensure that (1) permitted routes, such as those required by schedules with different treatment arms, could be defined and (2) the method could support controlling individual schedules dynamically (eg, as individual research participants were reviewed during their visits)

#### FHIR PlanDefinition

The Vulcan SoA IG [8] was used as the starting point for reviewing and evaluating methods to reflect the approaches mentioned earlier as FHIR resources. The primary resources used were *PlanDefinition, ActivityDefinition,* and the associated resources that are required to configure a complete description for a specific study (ie, *ReseachStudy, ResearchSubject,* etc). The main *PlanDefinition* elements investigated were *action.condition, action.relatedAction, action.timing*, and the 5 *action.<xxx>Behaviors*. All work was undertaken using version FHIR Release #5 (version 5.0.0) resources [1].

#### Model Testing and Proof of Concept

Graph database methods were used to develop and test the specification methods. Proof-of-concept example graphs were built using the Python generalized programming language [9], the NetworkX graph and network libraries [10], and the pandas data analysis library [11].

FHIR resource examples were generated using the Python fhir.resources library [12,13] and HL7 FHIR Shorthand definitions [13]. The yED graph editor (yWorks GmbH) was used to create the visual graph presentations and as an editor to create specific test examples [14]. The accuracy of the FHIR resources versus the graph model was confirmed by visual and programmed comparisons of the resulting definitions. The FHIR resources generated from each proof-of-concept example were confirmed as valid FHIR resources by loading them to publicly available FHIR end points, recovering the specifications using FHIR searches, and confirming that the full original specifications could be recovered without information loss.

#### SoA Example

Examples and illustrative figures used in this paper are based on the SoA graph shown in Figure 1.

### Ethical Considerations

The study does not include human participants, use of medical records, or patient information.

## Results

### Multiple SoA Paths

**Figure 2:**
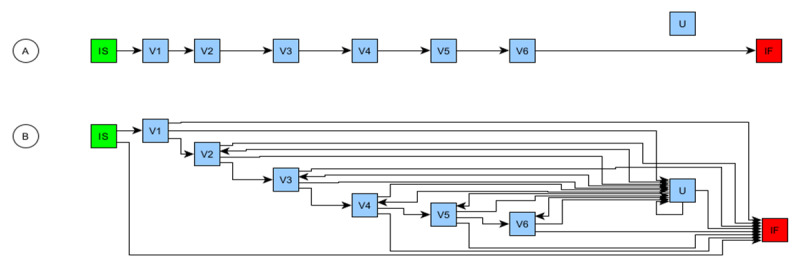
The schedule of activities directed graph representations of the visit schedule described in Table 1. (A) Protocol explicitly defined schedule with “floating” unscheduled visit (U). (B) Expanded version with all implied permitted paths defined. Two important implied paths are now present: routes for a participant to leave the study at any point (eg, V1>IF), and routes to and from U, if required. IS: instantiation start; IF: instantiation finish.

Figure 2 illustrates the general problem that arises even with a simple study design when considering how to define all protocol-defined permitted paths. Figure 2A shows how the scheduled visits of Table 1 might be presented as a directed graph. However, the “unscheduled” visit “floats” independently as it is to be used on an “as required” basis over the period of the planned visits.

**Figure 3:**
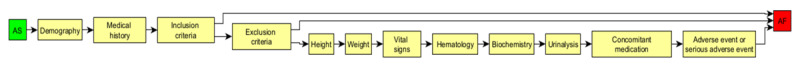
The schedule of activities directed graph representation of visit V1 activities from Figure 1, showing the protocol specified order (left to right) with the added requirements for those cases where (A) the inclusion criteria are not met, or (B) exclusion criteria are present. These paths formally define how to finish the visit “early.” The resulting visualizations, although busy, remain user-friendly from a review or quality control perspective. AS: activity start; AF: activity finish.

Defining all permitted paths (ie, extending Figure 2A to add all protocol-implied paths) is shown in Figure 2B and is easily achieved simply by adding appropriate visit-visit edges. Similarly, all potential activity sequencing options can be defined using the same method (Figure 3).

**Figure 4:**
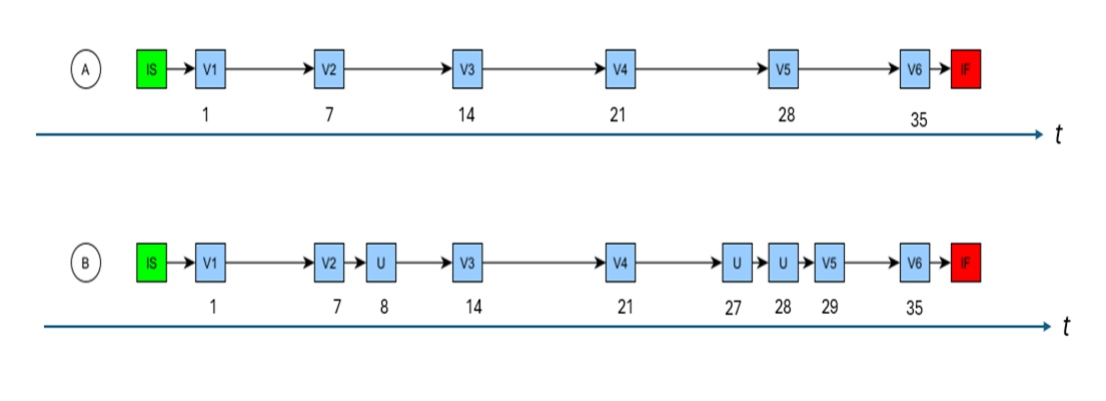
(A) Timing calculation attributes per protocol schedule, together with the relative day on which the visit occurred. (B) The same schedule but with 3 unscheduled visits. The first unscheduled visit (U; day D8) has no effect on the planned schedule, whereas the second (D27) and third (D28) U caused V5 to occur on D29 rather than on D28. In this case, the relative day of the visits cannot be determined from the planned timings alone. IS: instantiation start; IF: instantiation finish.

No additional node or edge attributes beyond those listed in Table 2 are required to define all permitted paths. However, ensuring that the timing calculations for any path are computable cannot be achieved using the planned timings alone. Here, the problem is that the scheduled planned timings are node (visit) attributes, but calculating the timings for any path requires a summation of the transitions along whichever path is selected (Figure 4).

Unscheduled visits may necessitate rescheduling of scheduled events. The addition of a *transitionDelay* edge attribute (Table 3; defined as the time to wait before transitioning to the next node) was found to provide the necessary timing information. It also provides the basis for a key timing consistency check.


*plannedTiming (V_n_)=sum(transitionDelay) from referenceTimepoint to V_n_*

**Table 3:** Edge attributes required for path timing calculations.

Edge attribute	Minimal required attribute	Notes or example
transitionDelay	Yes	“Wait” time before moving from source to target nodes (ie, V1>V2 7d)
transitionWindow	No	Permitted transitionDelay variance
transitionType	No (default)	start-to-start, default; start-to-finish; finish-to-start; and finish-to-finish

### Conditional Scheduling Attributes

Figures 2B and 3 also illustrate that there are many cases where specific conditions require different (but permitted) routes to be followed. Some cases are generic and are present in any study (eg, participant’s right to withdraw at any time), some are protocol-specific (eg, if the participant is male, a pregnancy test is not required), and some complex, as illustrated in Figure 2B for managing all “unscheduled” path options (eg, an “unscheduled” visit following V_n_ cannot return to early visits, only being able to proceed to the next planned visit).

To accurately carry all conditional scheduling requirements within a single SoA graph, it was found that 3 things were required as follows:

1. A method to specify (and therefore recognize) a graph within the graph.
2. A syntax to define how to select a subgraph and restrict access to identified paths in the graph.
3. A method that can be implemented as a dynamic graph (ie, as the graph changes over time).

Both graph node attributes and edge attributes in the model here could be used to hold conditional scheduling information. Adding conditions to the nodes (eg, [visit] *repeatAllowed: true/false*) was found to satisfy some standard requirements (eg, can a visit be repeated? *V2: repeatAllowed: false,* and *Unscheduled: repeatAllowed: true*), but the other requirements were very poorly satisfied (particularly regarding what routes are available following an unscheduled visit). Adding the following edge attribute was found to accurately and easily model all the SoA tested use cases:

- Attribute: transitionRule
- Minimal required: yes
- Explanation: rule specifications that can be resolved to True (path can be selected) or False (path unavailable)

This approach was also user-friendly, as only the conditions on each edge (transition) were needed to ensure the use of any specific path (ie, by answering the question “under what conditions can this path be selected or is not available?” and this can also include multiple conditions).

### Conditional Scheduling Syntax

A parameterized specification syntax was developed to hold the edge attribute conditions that can be consumed as inputs to a truth table engine that would resolve all edge conditions to the single “true” or “false” as described above. Simple *{“function”: “inputs”}* pairs were used to specify the “true” or “false” state at runtime for that condition (eg, *{“consentObtained”: true*}) or any combination of conditions.

The following examples show how several commonly required SoA conditional requirements can be defined using this approach.

### Dynamic Graph Specification

The primary use case here is the definition of permitted paths through the study, including, but not limited to, the primary path. For example, in Figure 2B, paths exist to and from “unscheduled” to all “visits” permitting return to the primary schedule. However, “unscheduled” visits cannot return to a previously visited “visit” (discussed earlier). Two rules are required for this case: one to confirm the existence of previously visited visits, and one to ensure that all “future” visits have not yet been visited. The following example shows the rules required on edge U>V3 in Figure 2B, which will ensure that only this path is available at runtime:

{‘interactions_exist’: [‘IS’, ‘V1’,’V2’]}, {‘interactions_not_exist’: [‘V3’, ‘V4’, ‘V5’, ‘V6", ‘IF’]}

(ie, If visits instantiation start [IS], V1, and V2 have occurred and visits V3, V4, V5, V6, and instantiation finish [IF] have not occurred, moving from unscheduled visit [U] to V3 is allowed).

Similar rule pairs, when applied to all U>V_n_ edges, can then ensure that only “forward” paths are available for selection.

### Conditional Activity Selection

Restricting, adding, or skipping activities dependent upon participant conditions is a very common feature of SoAs, with the details often provided as footnotes to SoA tables. Examples include requiring pregnancy testing if the participant is female (and conversely not requiring this if the participant is male) and not proceeding with further tasks if inclusion criteria cannot be met (Figure 3). Example rules for these cases are simple to define, but consideration of all edge options is usually required, as follows:

On edge to “exclusion criteria” {“if_criteria_met”:true}

On edge to “AF” {“if_criteria_met”:false}

### Conditional Repeats

An important feature of many SoAs is the requirement to repeat activities (eg, blood pressure measurements) or to repeat visit blocks. This last use case is particularly important in the case of many oncology studies where repeating treatment cycles is an inherent part of the study design and will have some limit on the number of cycles and the requirement to exit the study if too many cycles are proving necessary. Typical edge rules for controlling repeat or cycle situations include the following:

Repeat blood pressure measurement 3x. On edge to “self”: {“maxRepeats”:4}

*Limit the number of cycles to a max of 5.On edge to start of cycle*: {“n_cycles”: “<6”}

### Subject States, Milestones, and Events

Often, the requirement to select different SoA paths is dependent upon states, milestones, or events, as, for instance, defined by the FHIR ResearchSubject resource [1]. This can be illustrated by the following examples:

To enter an off-label extension study.

On edge to Open Label Extension schedule: {“continue_to_OLE”: true}

If an adverse event occurs.

On edge to adverse event activity: {“record_AE”: true}

### Multiple Conditions

The syntax also permits any number of conditions to be defined easily within a single SoA graph for any given edge to obtain a sample for genetic testing. This can be illustrated by the following example:

{“studyPhase”:”onStudy”}, {“sampleObtained”:”true”}, {“geneticTestingConsent”:”true”},

The runtime assessment of each condition then serves as the input to a logic engine that determines the final true or false state.

### Undefined SoA Graphs: Implied Edges

Undefined SoA paths can also be recognized using this approach. An undefined path is defined here as one that may occur but is not recognized formally within the SoA graph. A good example of this is the right to withdraw from a study at any time or skip an activity. Formally recognizing every point where these conditions may occur and providing a defined path may add too much complexity to the graph, and it may be unreasonable to model it. However, placing a general requirement on all transitions (edges) of *{“withdrawn”: false}* will permit proceeding through the schedule until *{“withdrawn”: false}* == “false” (ie, withdraw is now “true”). Using suitable runtime coding, the “undefined” transition can be applied to the specific participants’ SoA graph instance.

### FHIR SoA PlanDefinition Review

The primary objective of this study was to identify methods that enable SoAs to be more fully defined using FHIR resources. The SoA graph review identified the necessary additional requirements needed to satisfactorily model SoA specifications, such as those shown in Figure 1. To successfully define these specifications using FHIR resources, they must meet the following requirements:

1. Represent all SoA-identified paths
2. Recognize and “respond” to conditional cases
3. Allow consuming applications to be able to “walk” any permitted path

The Vulcan SoA IG [8] was used as the starting point for developing a more comprehensive SoA FHIR model with the goal of extending or modifying it to be able to manage the complex designs, conditions, and “variations on the theme” as discussed earlier.

### FHIR PlanDefinition SoAGraph Definition

From the graph attributes model developed earlier, it follows that for the *PlanDefinitions* to fully specify all SoA requirements, it needs to hold a definition of the SoA graph, and not the SoA table. Reviewing the use of the *PlanDefinition.action* and *PlanDefinition.action.action* elements, the basic graph node and edge relationship can be defined. Specifically, with nodes (visits) mapped to *PlanDefinition.actions* and edges(transitions) mapped to *PlanDefinition.action.actions,* all the relationships defined, for example, in Figure 1, can be specified within a single *PlanDefinition.*

**Figure 5:**
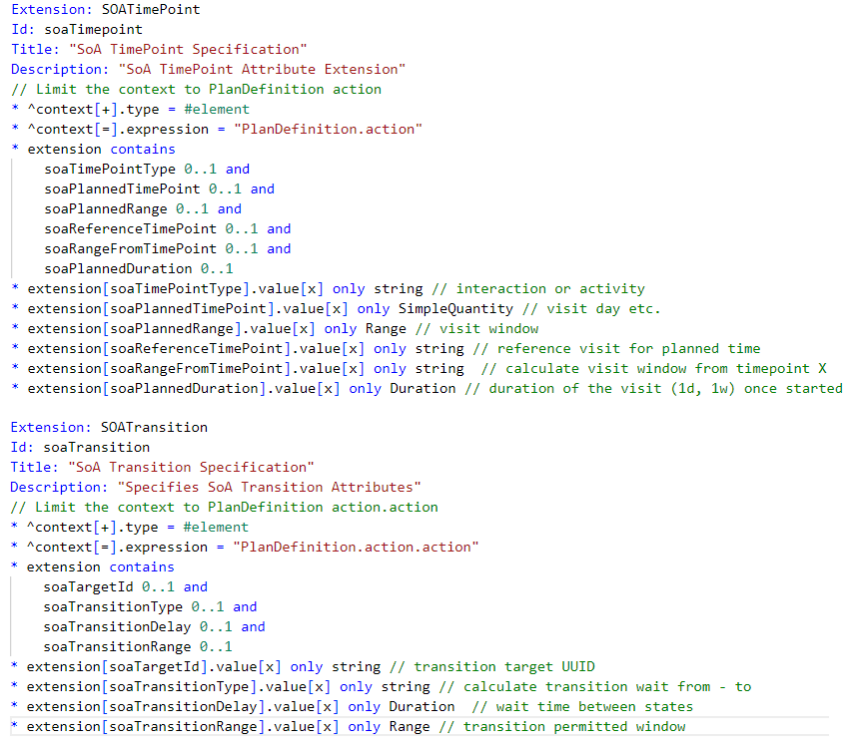
Fast Healthcare Interoperability Resources extensions (as FHIR shorthand) used to associate the soaGraph node and edge attributes with PlanDefinition.action (SOATimePoint) and PlanDefinition.action.action (SOATransition), respectively.

*PlanDefinition.action* in its standard form does not support those elements (attributes) to hold all the necessary SoA specification details. These have been added using 2 extensions, that is, *soaTimePoint* and *soaTransition* (Figure 5 [7]) to hold the graph information, which in turn then forms the basis for an *soaPlanDefinition* profile (Multimedia Appendix 1).

### FHIR PlanDefinition SoAGraph Condition and Selection Definition

**Figure 6:**
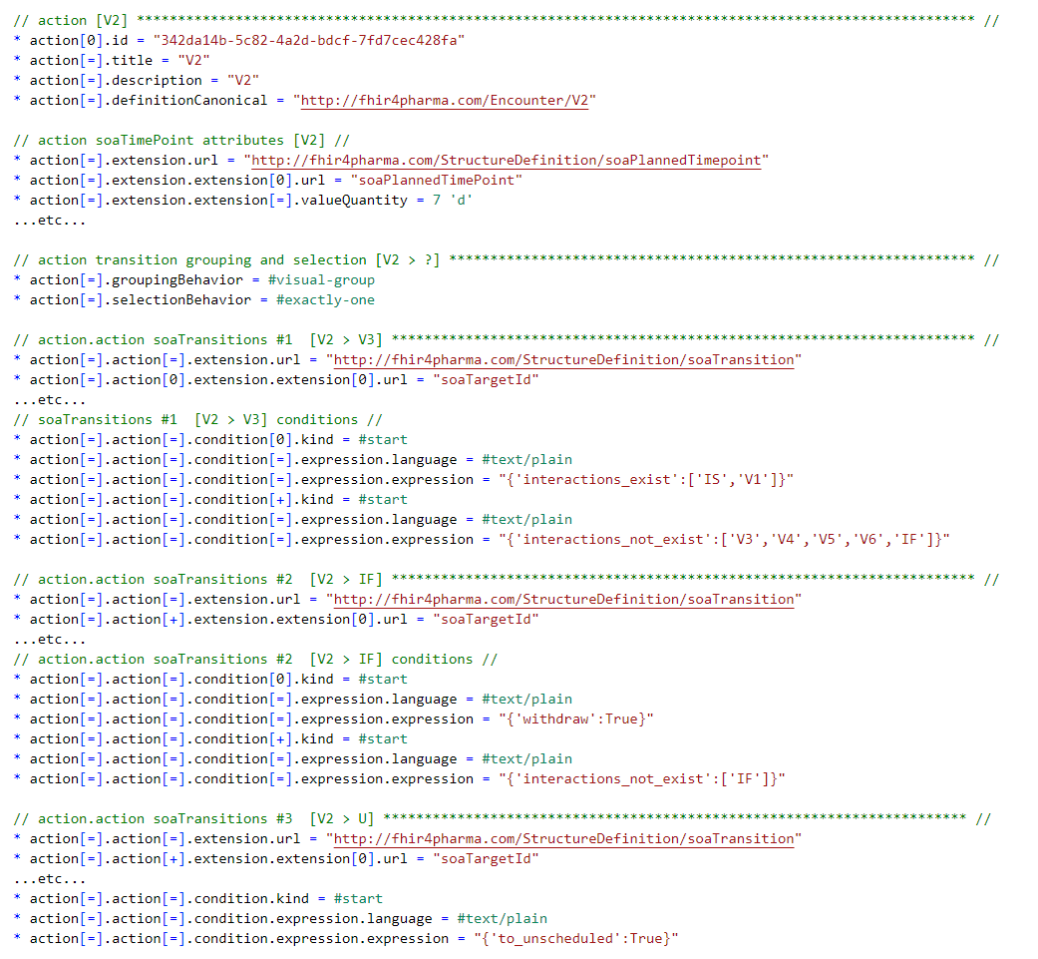
FHIR shorthand annotated version of PlanDefinition definition of visit V2 (.action) and its associated soaGraph transitions: V2>V3, V2>IF (instantiation finish; withdrawal), V2>U (unscheduled visit; .action.action). V2 attributes are defined using the ...action.soaPlannedTimepoint extension. The selection behavior is defined by ...action.groupingBehaviour and ...action.selectionBehaviour. For each path from V2, the conditions that must be met for that path to be available for selection are given in ...action.action.condition(s). V2, V3, U, and IF are the schedule of activities time points in Figure 2.

Conditional SoA requirements can now be specified using the *PlanDefinition.action* element *condition* (applied to *PlanDefinition.action.action*), with each edge having all or any true or false conditions for that transition. When combined with *PlanDefinition.groupingBehaviour* and *PlanDefinition.selectionBehaviour,* path selection can be restricted to 1 path only, with only those transitions that are permitted being available for selection. Figure 6 shows the FHIR Shorthand specification for node V2 in Figure 2 and Multimedia Appendix 2.

To operationalize the FHIR PlanDefinition within the workflow of a clinical system, such as an EHR, additional tooling is required. The illustrative syntax presented in the examples—for instance, *expression = “{‘interactions_not_exist’: [‘V3’, ‘V4’, ‘V5’, ‘V6’, ‘IF’]}”—*serves to demonstrate the underlying logic but is not directly executable within an FHIR environment. In practice, these expressions must be translated into an FHIR-compatible solution, typically by leveraging FHIRPath, clinical quality language, or system-specific functionality. Such translation ensures that the abstract representations in the PlanDefinition can be implemented as concrete, machine-interpretable instructions within the clinical system’s workflow.

### Proof of Concept

**Figure 7:**
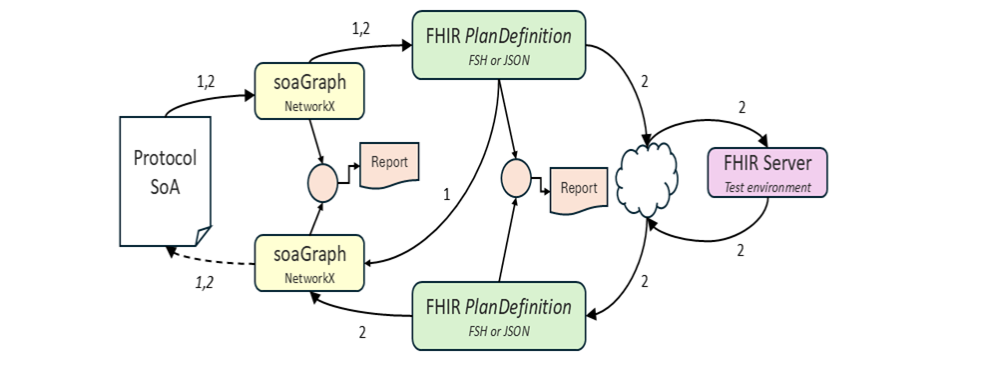
Proof-of-concept testing overview. The numbers highlight the 2 principal testing cycles used. Recovered products were compared with the original for reviewing structural equivalence and information loss throughout the development process. FHIR: Fast Healthcare Interoperability Resources; FSH: FHIR Shorthand; SoA: schedule of activities.

All the interim and final products, methods, and results described earlier were validated using the testing schedule shown in Figure 7. Once interim inconsistencies were reviewed and resolved, no SoA information loss was present after recovering an soaGraph from a FHIR *PlanDefinition* (Figure 7; test cycle 1) or after loading and recovery from test FHIR servers (test cycle 2).

**Figure 8:**
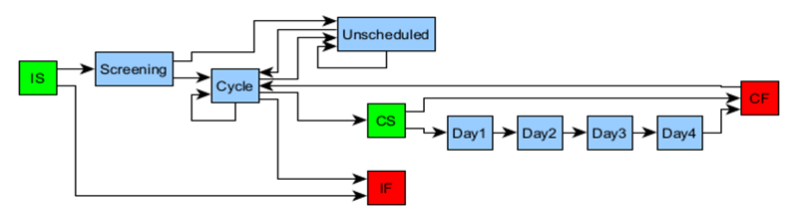
Template soaGraph for studies with cycles. Using appropriate edge attributes and conditions, it can be modified for various study designs. The green and red nodes are included to delineate the graph instantiation and the start and finish of the treatment cycle. CS: cycle start: CF: cycle finish; IF: instantiation finish; IS: instantiation start.

More than 25 studies, ranging from relatively simple designs, as shown in Figure 1, to complex studies incorporating cycles and multiple SoAs (not shown), were used to test and validate the approach. All tests could be accurately defined, with the most frequent finding during this exercise being that it highlighted inconsistencies in the protocol specifications themselves. It also lends itself to defining SoAs more succinctly and potentially more accurately. Figure 8 is a template for studies that includes cycles and shows that each required visit (interaction) is only defined once. With appropriate edge (transition) attributes and conditions, this can be modified to define many specific study scenarios.

## Discussion

### Overview

The HL7 FHIR standard is becoming, if not already, the de facto healthcare interoperability standard [2,15-18]. The primary source of many clinical trial data is EHRs, and studies usually require manual transcription of these data into electronic data capture systems. For both quality and volume reasons, this is often not optimal and has led to efforts to obtain data directly from electronic data capture systems (direct data capture) [19-22].

This study’s protocol and the SoA provide the primary definitional source for a study’s required research data and details of other operational requirements. The value of definitional FHIR resources to support clinical research and similar initiatives has been recognized in the Specialized Definitional Artifacts category [1].

The *PlanDefinition* resource is the primary resource for defining “a predefined group of actions to be taken in particular circumstances.” The Vulcan SoA IG [23] has developed a set of profiles using this resource to define SoAs. As mentioned earlier, this model does not include methods to define either conditional scheduling, certain study designs (notably cycles), or the scheduling of expected but not planned events (particularly “unscheduled visits”). This work has re-evaluated how *PlanDefinition* might be modified, revised, or extended to model these situations.

### Principal Findings

Using a systematic review of the relationships and attributes required to define the SoA characteristics discussed earlier, a graph-based method has been developed that can manage all these cases. Subsequently, by associating *PlanDefinition.action* and *PlanDefinition.action.action* directly with graph nodes and edges, respectively, SoAs with all scheduling variations, conditional paths, and methods to manage planned but unscheduled events can be defined within a single *PlanDefinition.* Recognizing that an SoA “activity” has both timing and task elements (ie, is “X” in tabular SoAs), the model formally disassociates the scheduling information from the task or activity definitions, which can then be linked using the *PlanDefinition.action.relatedAction.targetId* element. This approach also enables SoA activities as described in study protocols (or not described) to be fully traceable in the SoA schedule, but specified fully using other appropriate FHIR definitional resources (eg, *ActivityDefinition* and *ObservationDefinition*)

### Comparison With Prior Work

Earlier work based on using both graph methods for SoA definition [23,24] and FHIR for interoperability [25-27] has shown the value of this approach for communicating sponsor protocol requirements systematically for implementation in EHRs and similar systems [4,21,22,28-31]. The work here has re-evaluated the approach to specifying SoAs as FHIR *PlanDefinitions* to find solutions to several key required SoA characteristics not addressed previously. The model described here uses a radically different conceptual approach, redefining several *PlanDefinition* elements to be able to hold a graph representation of an SoA. The current method can successfully represent a wider range of SoA concepts than before and can also specify a large range of other SoA use cases that are key to many protocol designs (not shown). Because here all SoA requirements can be accurately specified within a single *PlanDefinition,* this should simplify the implementation by consuming applications. It is also clear that, with modification or the use of standard *PlanDefinition* elements, it can support other important SoA use cases not directly considered here (eg, protocol amendments with SoA consequences)

### Limitations

The methods in this study were developed using FHIR Release #5 (version 5.0.0) [1] and are not necessarily compatible with earlier FHIR versions.

### Conclusions

The methods described in this study offer an alternative approach to defining clinical study SoAs using FHIR definitional resources compared to previously published articles and may offer advantages with regard to some key requirements not addressed by other proposed approaches.

## Appendix

Multimedia Appendix 1 Schedule of activity PlanDefinition extensions (JSON).

Multimedia Appendix 2 Schedule of activity PlanDefinition Figure 2 example (JSON).

## Abbreviations

EHR: electronic health record
FHIR: Fast Healthcare Interoperability Resources
HL7: Health Level 7
IG: Implementation Guide
SoA: schedule of activities

## References

[1] Welcome to FHIR. HL7 Fast Healthcare Interoperability Resources.

[2] Pimenta N, Chaves A, Sousa R, Abelha A, Peixoto H (2023). Interoperability of clinical data through FHIR: a review. Procedia Computer Science.

[3] (2024). Real-world data: assessing electronic health records and medical claims data to support regulatory decision-making for drug and biological products. U.S. Food & Drug Administration.

[4] Chatterjee A, Pahari N, Prinz A (2022). HL7 FHIR with SNOMED-CT to achieve semantic and structural interoperability in personal health data: a proof-of-concept study. Sensors (Basel).

[5] Clinical electronic Structured Harmonised Protocol (CeSHarp). International Council for Harmonisation of Technical Requirements for Pharmaceuticals for Human Use.

[6] (2025). 2025 - 05 Vulcan utilizing the digital protocol (UDP). Confluence.

[7] Richardson A (2024). Representing clinical study schedule of activities as FHIR resources: required characteristic attributes. J Soc Clin Data Manag.

[8] (2023). Clinical study schedule of activities. HL7 International.

[9] Python.

[10] NetworkX.

[11] Pandas.

[12] fhir.resources 8.1.0. PyPI.

[13] (2024). FHIR shorthand. HL7 International.

[14] yEd - graph editor. yWorks.

[15] Saripalle R, Runyan C, Russell M (2019). Using HL7 FHIR to achieve interoperability in patient health record. J Biomed Inform.

[16] Mukhiya SK, Lamo Y (2021). An HL7 FHIR and GraphQL approach for interoperability between heterogeneous electronic health record systems. Health Informatics J.

[17] Monteiro SC, Cruz Correia RJ (2022). FHIR based interoperability of medical devices. Stud Health Technol Inform.

[18] Raso E, Loreti P, Ravaziol M, Bracciale L (2024). Anonymization and pseudonymization of FHIR resources for secondary use of healthcare data. IEEE Access.

[19] Aoyagi Y (2015). Direct data transfer from HIS (hospital information system) to sponsor for clinical trials. Proceedings of the 12th Annual Meeting DIA JAPAN 2015.

[20] Lombardo G, Couvert C, Kose M, Begum A, Spiertz C, Worrell C, Hasselbaink D, Didden EM, Sforzini L, Todorovic M, Lewi M, Brown M, Vaterkowski M, Gullet N, Amasi-Hartoonian N, Griffon N, Pais R, Rodriguez Navarro S, Kremer A, Maes C, Tan EH, Moinat M, Ferrer JG, Pariante CM, Kalra D, Ammour N, Kalko S (2023). Electronic health records (EHRs) in clinical research and platform trials: application of the innovative EHR-based methods developed by EU-PEARL. J Biomed Inform.

[21] De Moor G, Sundgren M, Kalra D, Schmidt A, Dugas M, Claerhout B, Karakoyun T, Ohmann C, Lastic PY, Ammour N, Kush R, Dupont D, Cuggia M, Daniel C, Thienpont G, Coorevits P (2015). Using electronic health records for clinical research: the case of the EHR4CR project. J Biomed Inform.

[22] Welker JA (2007). Implementation of electronic data capture systems: barriers and solutions. Contemp Clin Trials.

[23] Richardson A Approaches to schedule of activities (SOA) specification for automated implementation. PHUSE EU Connect 2020: ML05.

[24] Low G, Ward M, Richardson A, PHUSE Research on FHIR Project Team (2021). Study designs using FHIR: schedule of activities exchange using FHIR resources. PHUSE EU Connect 2021: RW05.

[25] Genyn P, Richardson A The role of FHIR Resources in ensuring semantic equivalence in EHR2EDC direct data capture. PHUSE EU Connect 2023: RE08.

[26] Genyn P, Richardson A The role of FHIR resources in testing and validating an EHR to sponsor data pipeline. fhir4pharma.

[27] Leroux H, Metke-Jimenez A, Lawley MJ (2017). Towards achieving semantic interoperability of clinical study data with FHIR. J Biomed Semantics.

[28] Coorevits P, Sundgren M, Klein GO, Bahr A, Claerhout B, Daniel C, Dugas M, Dupont D, Schmidt A, Singleton P, De Moor G, Kalra D (2013). Electronic health records: new opportunities for clinical research. J Intern Med.

[29] Garza M, Myneni S, Fenton Sh, Zozus Mn (2021). eSource for standardized health information exchange in clinical research: a systematic review of progress in the last year. J Soc Clin Data Manag.

[30] Laaksonen N, Varjonen JM, Blomster M, Palomäki A, Vasankari T, Airaksinen J, Huupponen R, Scheinin M, Juuso Blomster (2021). Assessing an electronic health record research platform for identification of clinical trial participants. Contemp Clin Trials Commun.

[31] El Emam K, Jonker E, Sampson M, Krleza-Jerić K, Neisa A (2009). The use of electronic data capture tools in clinical trials: web-survey of 259 Canadian trials. J Med Internet Res.

